# Attention allocation on mobile app interfaces when human interacts with them

**DOI:** 10.1007/s11571-021-09760-4

**Published:** 2021-12-27

**Authors:** Li Zhu, Gaochao Cui, Yan Li, Jianhai Zhang, Wanzeng Kong, Andrzej Cichocki, Junhua Li

**Affiliations:** 1 Computer & Software School, Hangzhou Dianzi University, Hangzhou, Zhejiang Province 310018 China; 2 Key Laboratory of Brain Machine Collaborative Intelligence of Zhejiang Province, Hangzhou Dianzi University, 310018 Hangzhou, China; 3 Department of Information System, Saitama Institute of Technology, Fukaya, Saitama 369-0293 Japan; 4 School of Art and Textile and Clothing Engineering, Changshu Institute of Techonology, Changshu, 215500 China; 5 Graduate School of Advanced Imaging Science, Multimedia and Film, Chung-Ang University, Seoul, 06974 Korea; 6 Skolkovo Institute of Science and Technology (Skoltech), Moscow, Russia 143026; 7 School of Computer Science and Electronic Engineering, University of Essex, Colchester, CO4 3SQ UK; 8 Laboratory for Brain-Bionic Intelligence and Computational Neuraoscience, Wuyi University, Jiangmen, 529020 China

**Keywords:** Advertising region, Attention allocation, Mobile app interfaces, Eye-tracking

## Abstract

With the popularity of smartphones and the pervasion of mobile apps, people spend more and more time to interact with a diversity of apps on their smartphones, especially for young population. This raises a question: how people allocate attention to interfaces of apps during using them. To address this question, we, in this study, designed an experiment with two sessions (i.e., Session1: browsing original interfaces; Session 2: browsing interfaces after removal of colors and background) integrating with an eyetracking system. Attention fixation durations were recorded by an eye-tracker while participants browsed app interfaces. The whole screen of smartphone was divided into four even regions to explore fixation durations. The results revealed that participants gave significantly longer total fixation duration on the bottom left region compared to other regions in the session (1) Longer total fixation duration on the bottom was preserved, but there is no significant difference between left side and right side in the session2. Similar to the finding of total fixation duration, first fixation duration is also predominantly paid on the bottom area of the interface. Moreover, the skill in the use of mobile phone was quantified by assessing familiarity and accuracy of phone operation and was investigated in the association with the fixation durations. We found that first fixation duration of the bottom left region is significantly negatively correlated with the smartphone operation level in the session 1, but there is no significant correlation between them in the session (2) According to the results of ratio exploration, the ratio of the first fixation duration to the total fixation duration is not significantly different between areas of interest for both sessions. The findings of this study provide insights into the attention allocation during browsing app interfaces and are of implications on the design of app interfaces and advertisements as layout can be optimized according to the attention allocation to maximally deliver information.

## Introduction

If computer is a crucial revolution that significantly changes the manners of life and work, phone is an evolution that makes easy access to cyber world and facilitate portable use. People can perform a number of tasks, such as information retrieval, communication, entertainment, healthcare and schedule management, by a phone without restrictions of location and time. A critical factor of expanding the use of smartphone is the increase in the number of mobile apps. In recent years, the number of mobile apps was incredibly increased and is still rapidly growing. According to the information of the statistics portal website^1^, there are 3.48 million available apps in the Google Play Store and 2.22 million apps in the Apple’s App Store as of the first quarter 2021. However, the number of apps in the Google Play Store is only 1.9 million as indicated in a paper published in 2016 (Allix et al. 2016). The statistics in 2021 shows that the most popular category in the Apple’s App Store is games, which is followed by the business and education categories^2^. These apps provide diverse services and functions to satisfy users of smartphone. To date, almost all software running on the platform of computer have equivalent or resemble versions that can be run on the platform of smartphone. However, the reverse is not the case. Some apps running on the platform of smartphone do not have an edition for the platform of computer. As we know, the screen size of the smartphone is much smaller than that of a computer, so the layout of app interfaces and information arrangement are very important to app developers when they design and develop an app (Khalid et al. 2015). These also affect user experience and then determine acceptance rate of an app. For instance, commercial advertisements are usually embedded into a free app and occasionally shown while a corresponding service or function is being provided to users from this app. People used to ignore banner-like information presented in highly contrasting images with bright colors, which is known as banner blindness (Muñoz-Leiva et al. 2019). To reduce or eliminate negative consequence of the banner blindness, advertisements should appear at proper place and time as well as being cohesive to the contents in order to maximize the effect of marketing. Towards this end, developer should first know how people allocate their attention to app interfaces. However, little is known currently and there is as yet no literature that directly addresses this question.

Human attention orientation is manifested through eye movements (van der Wel et al. 2018; Hunt et al. 2019; Rayner 2009). Longer gaze time (i.e., fixation duration) reflects higher interest towards that they are staring at. The data recorded by an eyetracker are analysed to obtain insights into humans’ attention or interpreted into an intention for interacting with outside world and has been successfully applied to a number of monitoring and assessment researches Misthos et al. 2018; Bodala et al. 2016; Ahonniska-Assa et al. 2018; Lander 2017; Chynał et al. 2012). One work is the investigation of preferred reading regions of a desktop computer screen (Buscher et al. 2010). In this work, an eye-tracker was utilized to record eye movements while university students browsed web pages for either retrieving computers with certain specifications or retrieving information matching predefined topics. They found that students allocated more attention to the middle viewing strip of the screen with an expansion to the upper and bottom edges at the left end of this strip. Another work is about interface evaluation (Goldberg and Kotval 1999). In this study, well-organized interfaces were compared to randomly-organized interfaces. Authors concluded that the shorter scan-paths and smaller covered area of these paths were observed in the case of well-organized interfaces, suggesting that well-organized interfaces resulted in more efficient search outcome. Kunze and his colleagues inferred language levels of participants based on the behaviour of eye focus (Kunze et al. 2013). They assumed that longer eye fixations were caused by unknown words and the higher occurrence rate of fixations implied lower level of language ability. This occurrence rate was negatively correlated with the score of a standardized English test of TOEIC (Test of English for International Communication). Attention can not only be tracked but also be predicted as demonstrated in a recent study (Steil et al. 2018). Eye gaze of the collaborator was also tracked and presented to a local worker for the purpose of assistance (Higuch et al. 2016) in a remote collaboration scenario. This study proved that eye-gaze based remote collaboration is feasible.

Evaluations derived from the eye-tracker data would be quantitative and reliable (Bruneau et al. 2002). This advantage is crucial for investigating human attention allocation to different parts of an app interface in this study. Therefore, we employed the eye-tracking technique to track human attention in the sessions. A review paper stated that visual perception was affected by a variety of cognitive factors including emotion and expectation (Vetter and Newen 2014). Attention is driven by both top-down cognitive supervision and bottom-up visual perception, which was frequently investigated with stimuli (Moore and Zirnsak 2017). In the context of target-free app browsing on a smartphone, attention allocation, to the best of our knowledge, has never been explored before our study. As layout and color are two features mostly relevant to human attention (Olivers et al. 2006), we thus modulated these two features to design an experiment with two sessions to investigate attention allocation during app browsing. For the session 1, original interfaces extracted from apps downloaded from the Apple Store were presented to participants. In this case, both layout and color features were included. In the second session, colors and background were removed to exclude the effect of color feature. This allowed us to inspect how attention allocation was linked to the layout alone. In addition, a study of the visual processing revealed that visual system differently responded to familiar and unfamiliar faces (Collins et al. 2018), which inspired us to explore whether the familiarity of the use of a smartphone is associated with attention allocation. We therefore investigated the correlation between the operational level of a smartphone and fixation time.

## Methodology

### Participants and operation level assessment

**Fig. 1:**
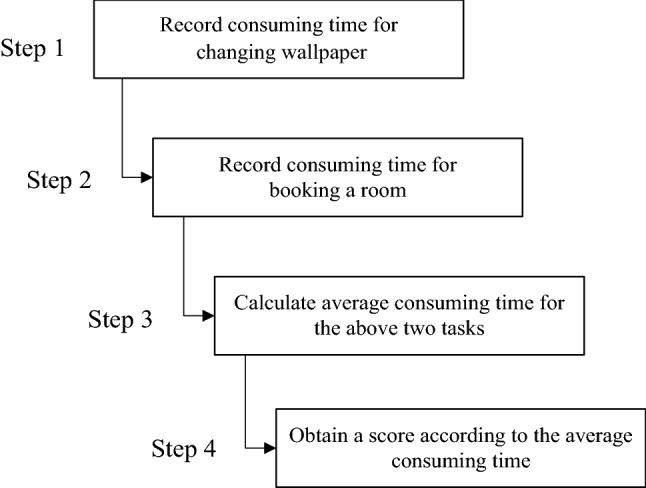
The calculation procedure for the score of operational level

We recruited 18 participants (male/female:10/8) in this study. They were undergraduate or graduate or doctoral students and have no history of eye diseases. Eight of them were fresh undergraduates from Republic of Korea with elementary Chinese. The rest of participants are Chinese without any knowledge of Korean. All have normal vision or corrected-to-normal vision. The average age is 25.1, ranging from 18 to 33. Each participant underwent two phone operating tests, namely changing wallpaper and booking a room through an app. The app used for the task of booking a room is Ctrip (in Chinese) for Chinese participants and Airbnb (in Korean) for Korean participants. The test of changing wallpaper aims to assess the skill in phone setting while the test of booking a room is used to measure the ability in the use of an app. Almost all people ever performed such both tasks but not frequently performed them in their daily life, which leads to an appropriate assessment of smartphone operation level without a bias caused by either ignorance or familiarity. The time spent on the completion of each task was recorded and averaged across two tasks. The highest score 6 was given if the average time is less than half a minute and the lowest score 1 was assigned when exceeding 50 s. These two time bounds were set to group people who perform either extremely fast or extremely slow and should be at the same level in smartphone operation. Time period from the 30th second and the 50th second was evenly divided into 4 intervals, corresponding to scores from 5 to 2, respectively. This score was referred to as operation level of smartphone (see the procedure in Fig. 1). The average operation level of smartphone across all participants is 3.9 (see Table 1 for respective participants).

### APP interfaces

There are two versions in the Apple Store and Google Play Store, respectively, for most of apps, especially for popular apps. These two versions are identical from functions to appearance. We, therefore, downloaded all apps used for this study from the Apple Store. Apps’ interfaces were then extracted to be images. All these images were assembled in a random order and sequentially presented to the participants in the sessions. In order to eliminate potential bias, we selected the interfaces with approximately even distributions in color, layout, icon and so on for four quarters (i.e., the upper left, upper right, bottom left, and bottom right areas). In order to eliminate the effect of linguistic context on the attention allocation, the participants are almost illiterate in the language shown on the interfaces. To this end, Korean participants browsed the interfaces in Chinese and Chinese participants were given the interfaces in Korean for browsing.

**Table 1:** Smartphone operational level for each participant

Participant	Operational level
1	1
2	5
3	6
4	6
5	4
6	1
7	4
8	4
9	5
10	3
11	5
12	4
13	4
14	3
15	5
16	4
17	3
18	4

### Eye-tracking system

A mobile device stand (MDS) was used to accommodate eyetracker and smartphone. Tobii Pro X2-30 was mounted in the slot located at the lower part of the MDS, while smartphone was fixed on the rack located at the middle of the MDS. A camera was on the top, facing perpendicularly to the screen of smartphone. The MDS can be adjusted to fit participants’ height and viewing angle. We made the distance from a participant’s eyes to the eye-tracker be within the range of 60-65 cm. Calibration was used to make sure that eyes can be correctly tracked. During the calibration process, participants were asked to look at points of the calibration grid. After the calibration, verification was performed to confirm whether the calibration is satisfied by checking whether the detected point is matched with the point the participant was asked to look at. This calibration was done separately for each participant. Tobii Pro X2-30 tracked eye movements at the sampling rate of 30 Hz.

### Experiment

**Fig. 2:**
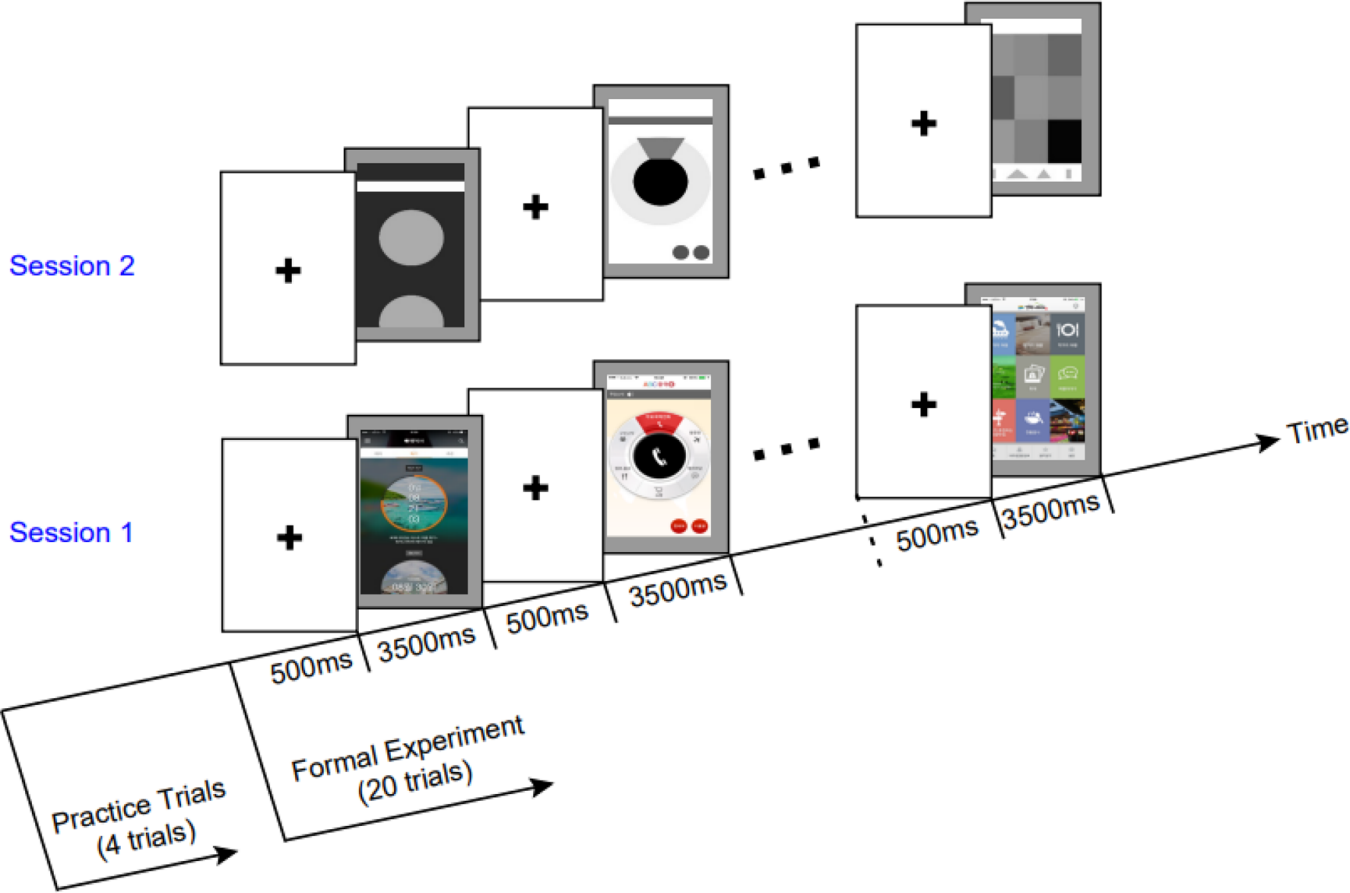
The protocol of experiment. Original app interfaces are used in the session 1, on which there are colors and background. In the session 2, all colors and background are removed and only layouts in gray-scale are retained. There are four trials used for the practice before the formal session with 20 trials. Each trial is comprised of a 500 ms period showing fixation cross and a 3500 ms period displaying app interface

There were two sessions conducted in this study. The original app interfaces were used in the session 1, on which there were colors and background. In the session 2, all colors and background were removed and only layouts in gray scale were retained. The sequence of interfaces displayed to the participants was the same to that in the session (1) The order of sessions was counterbalanced. That is, the half number of participants first performed the session 1 and the other half first performed the session (2) There was a short interruption between sessions to set up the next session. The protocol is illustrated in Fig. 2.

Participants sat in a comfortable chair with appropriate distance and viewing angle to a smartphone, and suitable screen brightness and contrast. All these settings were individually adjusted for each participant so that they can use the phone in familiar manner. This can diminish potential disturbance derived from unfamiliar phone use. A chin-rest was employed to minimize head movements of participants. This also helped improve attention focus recording by the eye-tracker. The smartphone is presented in its vertical orientation since mostly people use the mobile app interfaces vertically when human interacts with them and only for some special cases, such as watching videos, playing games, people would use the mobile app interfaces horizontally. Moreover, the interfaces with approximately even distributions in color, layout, icon and so on for four quarters were used, therefore, the orientation of the smartphone should have small impact on the attention allocation. During the sessions, participants browsed app interfaces that were automatically presented according to the protocol illustrated in Fig. 2. Participants did not need to switch interfaces by themselves and no action or response (e.g., pressing buttons) was required during browsing. The display of app interfaces was in a trial design. Each trial comprised a fixation cross display and an app interface display on the phone screen (see Fig. 2). In each trial, the fixation cross was shown for a period of 500 ms and followed by the display of the app interface lasting 3500 ms. There is no interval between trials. Participants were allowed to practise four trials before the formal session was started. After the practice, 20 trials (the interfaces used differ from the interfaces in practice) were performed in each session.

These experiments were reviewed and approved by Institutional Review Board of the Saitama Institute of Technology (No. 2018-01), following the principles outlined in the Declaration of Helsinki. All participants provided their written informed consent forms before engaging in the sessions.

### Data analysis

Attention fixation durations were extracted from the recorded eye-tracking data. Two metrics (first fixation duration and total fixation duration) were calculated based on these fixation durations. First fixation duration of one region represents how long a participant stares at this region at the first time. Total fixation duration is the sum of time spent on staring at a region throughout a trial. We evenly divided the whole screen of smartphone into four areas of interest (AOIs): upper left AOI (marked as AOI1), upper right AOI (AOI2), bottom left AOI (AOI3), and bottom right AOI (AOI4). Analysis of variance (ANOVA) was performed to evaluate whether or not there were significant differences between four AOIs in the total fixation duration and the first fixation duration. The post-hoc paired t-test was further conducted if there was a significance at the ANOVA analysis. Moreover, we also compared fixation durations between upper half part and bottom half part of the screen, and between left half part and right half part. In addition, we conducted correlation analysis between fixation durations and the operation level of smartphone.

## Results

### Total fixation durations for AOIs

**Fig. 3:**
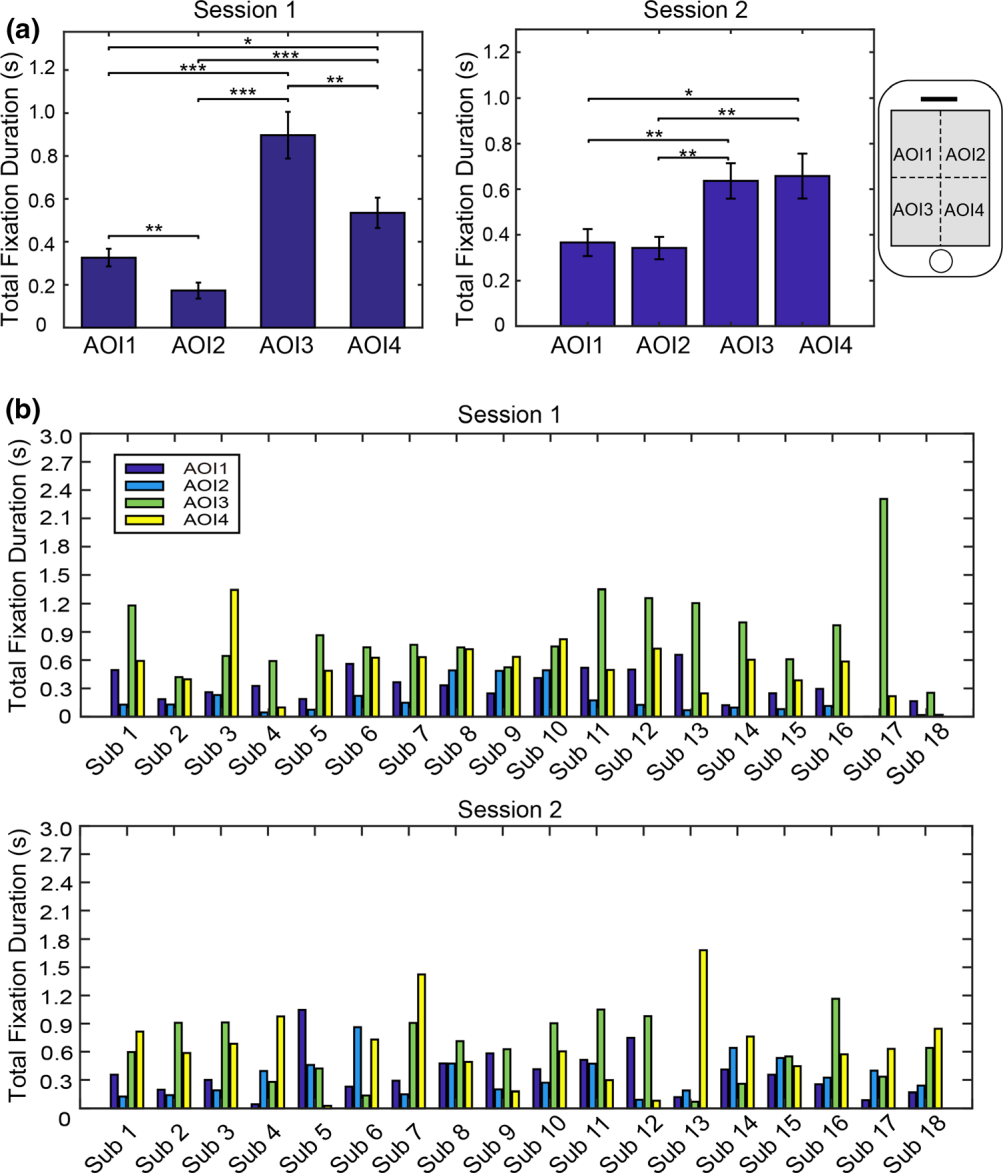
Comparisons of the total fixation duration between areas of interest (AOIs). **a** Average and standard errors of total fixation durations. Session 1: App interfaces with colors and background were used for browsing. Session 2: App interfaces without colors and background were used for browsing. Asterisks represent statistical significance levels (* p<0.05; ** p<0.01; *** p<0.001). **b** Total fixation durations for each participant and each AOI. Notes: missing bars mean the duration is 0 for those AOIs

According to the ANOVA analyses, the average total fixation durations are significantly different between four AOIs for both session 1 [F(3,68) = 19.78, p < 10^−8^] and session 2 [F(3,68) = 5.33, p < 0.005] (see Fig. 3(a)). The post-hoc paired t-test revealed that the total fixation duration was significantly different among AOIs in the session1, reflecting varying attention focus over different regions of the screen when participants browsed app interfaces with colors and background. When the colors and background were removed, the significant differences only exist between the upper part and bottom part. The durations were comparable between AOI3 and AOI4, as well as between AOI1 and AOI2. Figure 3(b) illustrates total fixation durations for each participant. It can be seen that the majority of participants are of the same distribution of attention oriented to the four AOIs.

**Fig. 4:**
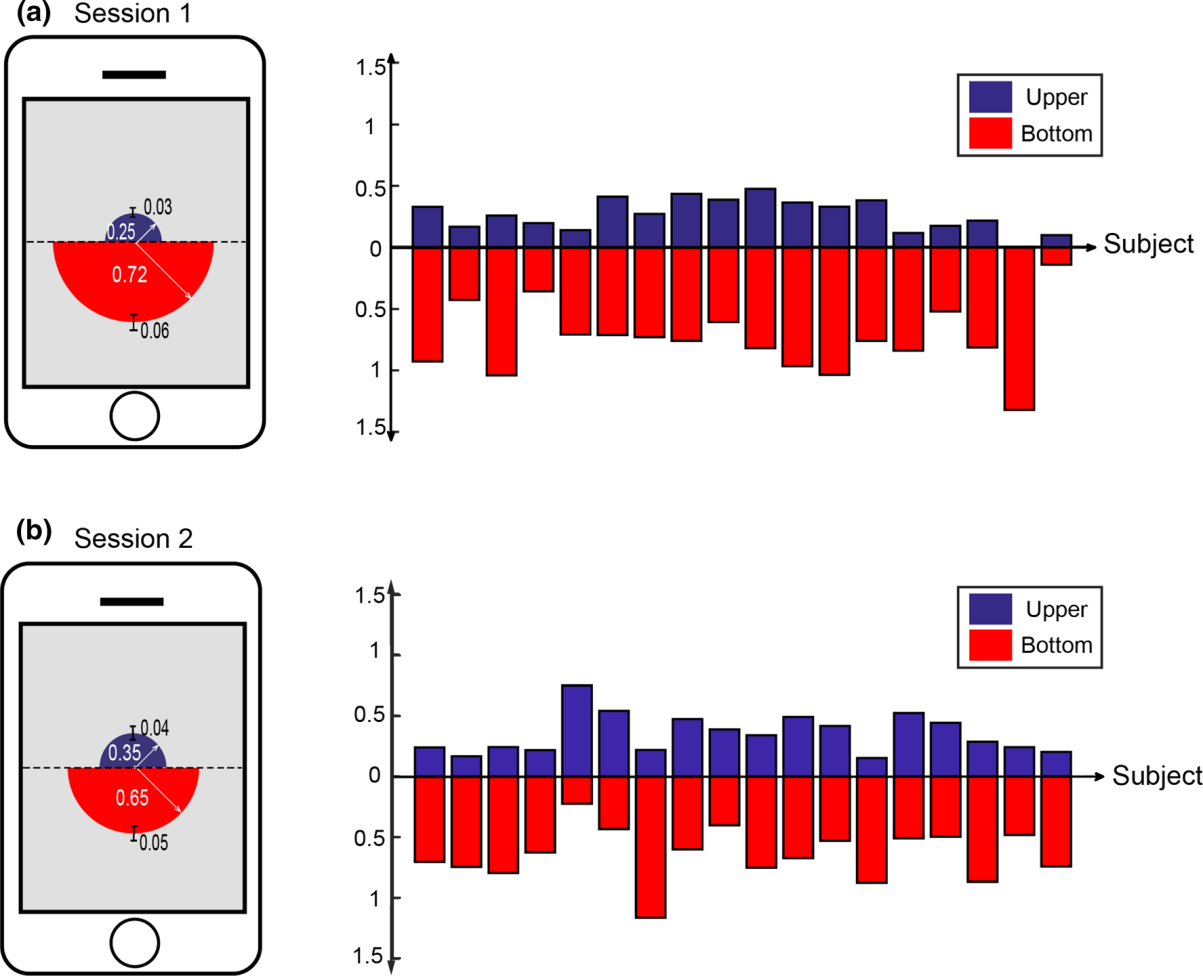
Total fixation durations of the upper and bottom half parts of the screen for the session 1 (shown in the panel (**a**)) and the session 2 (shown in the panel (**b**)). The means and standard errors of the total fixation durations averaged across participants are illustrated at the left side. Individual total fixation durations for each participant are shown at the right side

### Total fixation durations for halves

**Fig. 5:**
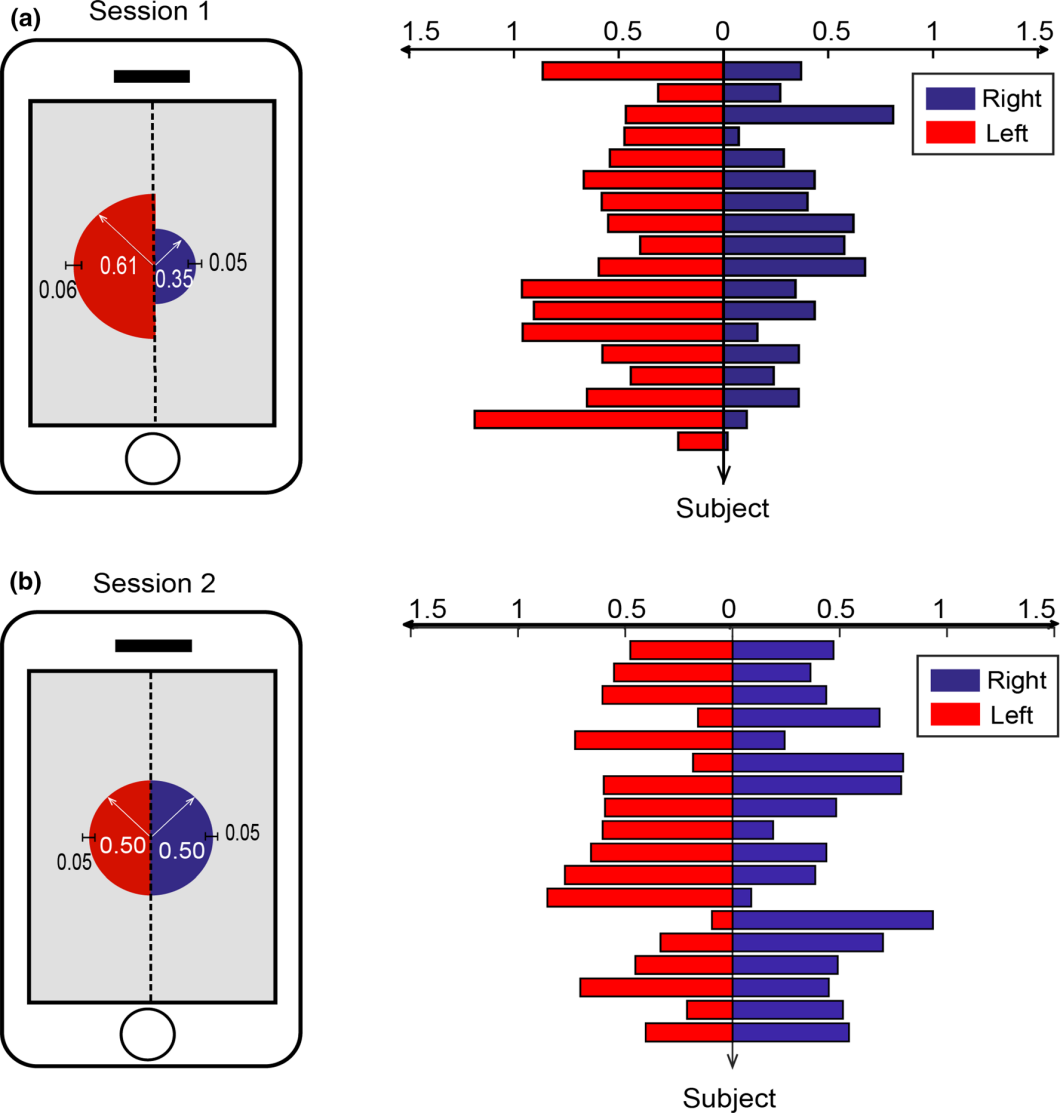
Total fixation durations of the left and right half parts of the screen for the session 1 (shown in the panel (**a**)) and the session 2 (shown in the panel (**b**)). The means and standard errors of the total fixation durations averaged across participants are illustrated at the left side. Individual total fixation durations for each participant are shown at the right side

Figure 4 depicts the average total fixation durations for upper and bottom areas of the screen, as well as individual durations. In the case of session 1, the average total fixation durations are 0.72 s and 0.25 s for the bottom area and the upper area, respectively. Their durations are significantly different [t(17)= −7.05, *p* < 10^−5^]. This phenomenon of the bottom-greater-than-upper is also observed in the session 2 (0.65 s versus 0.35 s, [t(17)= −3.53, *p* < 0.005]).In the comparisons between left half area and right half area of the screen, we found that the total fixation duration spent on the left half screen was significantly longer than that spent on the right half screen [0.61 s versus 0.35 s, t(17) = 3.25, *p* < 0.005] in the session 1 (see Fig. 5). There is no significant difference in the total fixation duration for the session 2 (0.50 s versus 0.50 s, *p* > 0.05).

**First fixation durations and the ratio of the first fixation duration to the total fixation duration**

**Fig. 6:**
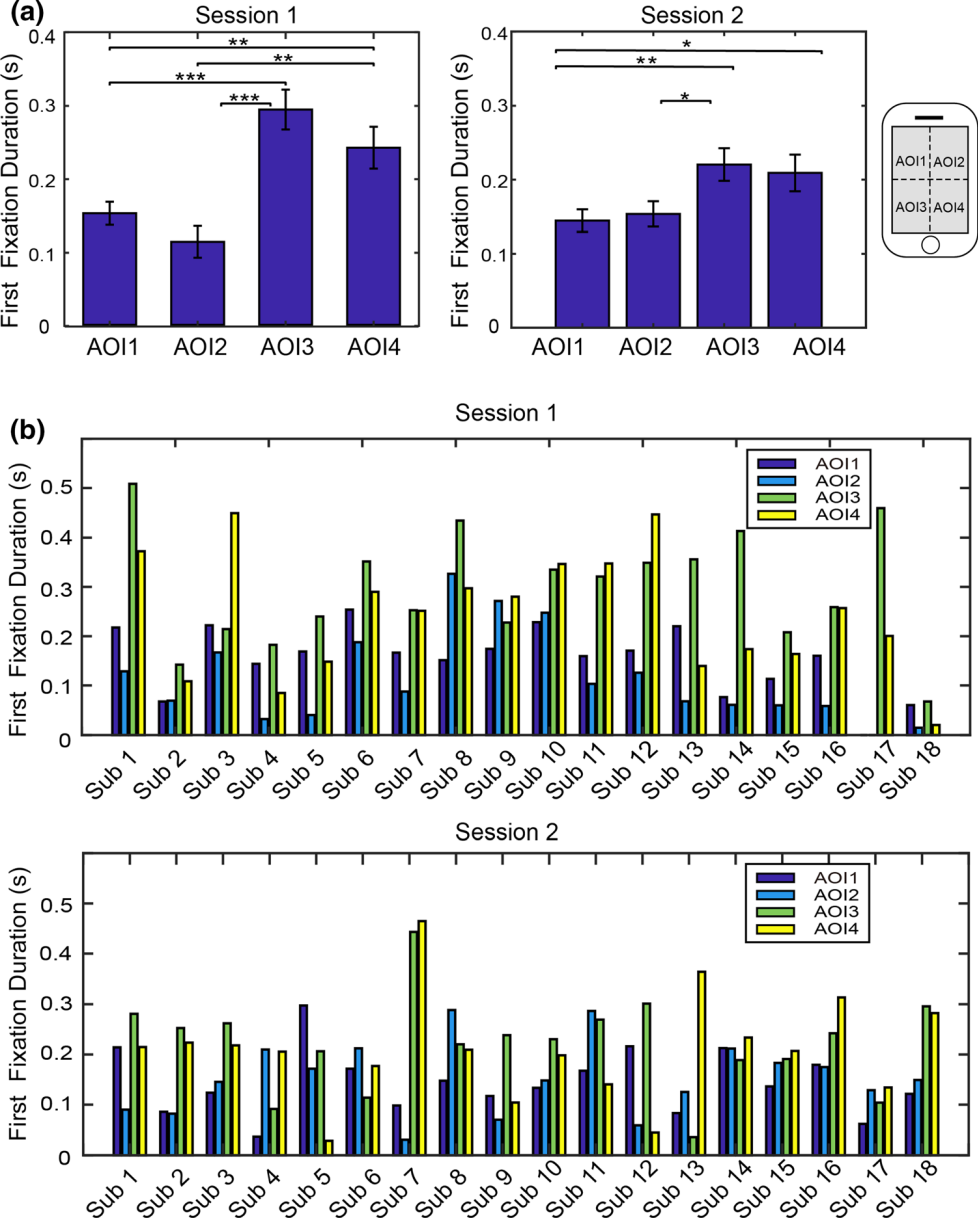
Comparisons of the first fixation durations averaged across participants. **a** Average first fixation durations. Session 1: App interfaces with colors and background were used for browsing. Session 2: App interfaces without colors and background were used for browsing. Asterisks represent statistical significance levels (* *p*<0.05; ** *p*<0.01; *** *p*<0.001). **b** First fixation durations for each participant and each AOI. Notes: mussing bars mean the duration is 0 for those AOIs

**Fig. 7:**
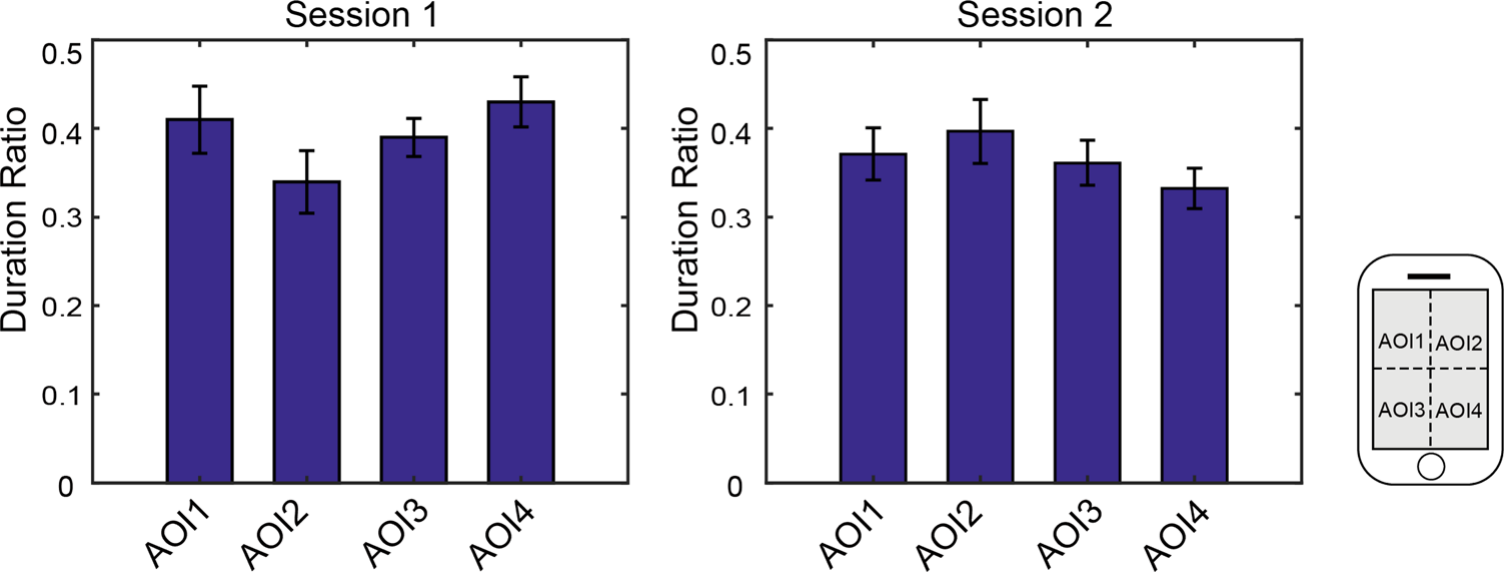
Comparisons in the average ratio of the first fixation duration to the total fixation duration

The results of the first fixation duration is very similar to the results of total fixation duration. There is significant difference between four AOIs in both session 1 [F(3,68) = 11.90, *p* < 10^−5^] and session 2 [F(3,68) = 3.61, *p* < 0.05]. The first fixation durations of AOI3 and AOI4 are significantly longer than that of AOI1 and AOI2 (see Fig. 6(a)). Duration difference between AOI1 and AOI2 is not significant. It is also not significant between AOI3 and AOI4 in duration difference. This leads to a significant difference between upper half and bottom half, and no significant difference between left half and right half. For the session 2, significant differences are found between AOI3 and AOI1, between AOI3 and AOI2, as well as between AOI4 and AOI1. All other pairs do not have significant differences. According to the results of ratio exploration, the ratio of the first fixation duration to the total fixation duration is not significantly different between AOIs for both sessions (see Fig. 7).

### Correlation between duration and phone operation level

**Fig. 8:**
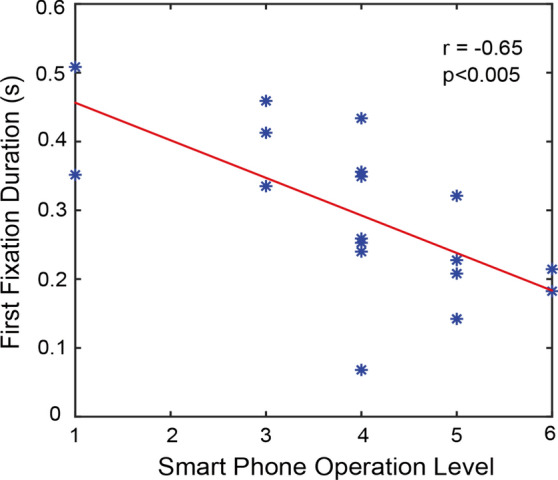
The correlation between the first fixation durations and smartphone operation level. Asterisks represent participants. The red line shows the best linear fitting

Because the AOI3 and AOI4 attracts dominant attention (see Fig. 3(a) and Fig. 6(a)), we further explored their correlations to the smartphone operation level. We found that the first fixation duration of AOI3 is negatively correlated with the smartphone operation level in the session 1 (see Fig. 8). The correlation extent is −0.65 with a statistical significance level of *p* < 0.005. There is no significant correlation between AOI4 and operation level. For the session 2, no significant correlation was found for both AOI3 and AOI4.

## Discussions

We explored human attention allocated to different areas of app interfaces when people browsing interfaces on a smartphone screen. The sessions revealed that participants did not evenly allocate their attention to the different areas of app interfaces. The bottom left area attracts more attention compared to other areas when participants browsed app interfaces with colors and background. Participants paid more attention on the bottom half part of screen, regardless of the presence of colors and background. However, attention allocation to the left and right half parts is related to the presence of colors and background, showing the left half part attracts more attention when browsing app interfaces with colors and background. Moreover, we found significant correlation between the first fixation duration of AOI3 and smartphone operation level when browsing app interfaces with colors and background.

The relative position between the eye and fixation point does not negatively affect the results because after the calibration, verification was performed to confirm whether the detected point is matched with the point the participant was asked to look at, which ensures that the participant could browse the whole mobile interface thoroughly. And the cross fixation is used for indicating the participant the onset of next trial and eliminating the effect of last trial such as the eye position readjusting. Moreover, the mobile device is fixed and the height of chair is adjusted to fit participants’ height and holding eyes’ viewing angle. All these settings were adjusted for each participant so that they can use the phone in familiar manner.

### Relationship between attention allocation and colors and background

As indicated in our results, colors and background on app interfaces affected the attention allocation. According to the session 1, participants were prone to pay more attention on the bottom left area when they browsed app interfaces with colors and background. When colors and background were removed from the app interfaces, participants paid comparable attention on the bottom left area and bottom right area. This finding might imply that attention allocation could be partially related to the neural processing of color and background perception, which is in agreement with the conclusion of attentional modulation of color processing in a previous neuroscience study (Anllo-vento et al. 1998). Although the colors and background affected the attention allocation to the left and right half parts of the screen, they do not affect the attention allocation to the upper and bottom half parts. In both sessions, we observed significantly longer fixation duration spent on the bottom half part compared to the upper half part.

### Link between first fixation duration and total fixation duration

In the case of app interfaces with colors and background for browsing, the proportion of the first fixation durations spent on different AOIs resembles that of the total fixation durations. The longer total fixation duration was observed on the AOI with longer first fixation duration. This is also tenable for the case of browsing app interfaces without colors and background (see Figs. 3(a) and  6(a)). This resulted in no significant differences between AOIs in the ratios of the first fixation duration to the total fixation duration (see Fig. 7). This finding suggests that participants were prone to repeatedly pay more attention on the AOI that they paid more attention at the first time. It signifies that the first impression is critically important, which should be taken into consideration while designing an interface or advertisement.

### Correlation between fixation duration and phone operation level

Participants with the higher phone operation levels gave shorter first fixation duration on the dominant AOI3 when browsing app interfaces with colors and background, resulting in negative correlation. This finding is intuitive. The person who possesses the higher phone operation level can shorten the fixation duration to obtain equivalent information, for which a person with the lower operation level needs to spend longer time. Nonetheless, we did not find significant correlation between total fixation duration and phone operation level. This might be due to the fixed time duration (3500 ms) for browsing each app interface, which might lead to that participants with the higher phone operation level have to spend all browsing time even they can obtain all information on the interface within shorter time period. When app interfaces without colors and background were used for browsing, no significant correlation was observed in both first fixation duration and total fixation duration. In this case, app interfaces were very concise and do not have much information for browsing. Therefore, the higher phone operation level seems to be useless. The participants with the lower phone operation levels can also obtain all displayed information. The remaining time would be spent for unintentionally and randomly browsing. This finding signifies that the amount of content shown on the interface should be appropriate to targeted customers. For instance, it might be suitable to give less content to the older population.

### Limitation and consideration

In this study, we used the interfaces which extracted from apps downloaded from the Apple Store, rather than experiment-oriented artificial interfaces. This empowers the used interfaces to represent app interfaces in the market and makes the findings in our study be able to have maximal generalization. However, the layout and distributions of properties (e.g., color) were varied across app interfaces. In this study, we selected the interfaces with approximately even distributions in properties to control the variation. The remaining variation across the interfaces should not overturn the findings because the variation is heterogeneous. For example, the area that has more colourful on one interface might have less colourful in another interface. This study explored attention allocation by dividing the whole screen of a smartphone into four areas equal in size, as well as halves. More divisions (e.g., 9 equal-sized areas) could be employed to investigate attention allocation. However, given the screen size of a smartphone is much smaller than that of a computer monitor, more divisions could lead to less precise capture of attention focus to each division because of the limited precision of the eye-tracker. Another point we want to mention is about the tasks used for assessing operational level of participants. The task used for the assessment is not unique. Other analogous tasks such as changing the ringtone could also be used. Due to the lack of the standards of operational level assessment, we proposed two kinds of tasks, which assess the skill of phone setting and the ability of the use of apps, respectively. In the future, the standards of operational level assessment are required to eliminate any potential bias. Due to the limited number of participants, our study serves as a preliminary exploration in attention allocation during browsing app interfaces, which had not yet explored prior to our study. The objective of the study is to reveal attention distribution when browsing app interfaces. We strived to eliminate interference factors such as contents and layout so that the findings were not due to those interferences. As we explored the attention distribution, the findings could inform the development of app interfaces. However, we did not claim that the findings in this study were the unique reference for designing app interfaces or advertising, or the method used in this study was the best one for designing app interfaces or advertising. This study served to reveal attention distribution as the purpose. Besides this purpose, it may provide useful information which can be taken into consideration when designing app interfaces or advertising. For example, more important information or contents could be arranged in the area that people pay more attention so that they can be read in a higher chance. The interface complexity should be compatible to the smartphone operation level of the targeted users. A pop-up banner with abundant information should be displayed longer for the users with low operation level compared to those who have the high level in the smartphone operation.

## Conclusions

In this study, we explored human attention allocated to different areas of app interfaces when people browsed app interfaces with and without colors and background. The experiment in our study demonstrated that people deployed different amounts of attention to different areas of app interfaces. Colors and background affect the allocation of attention towards different areas of app interfaces. The persons with higher operation level of smartphone are prone to spend less time for the first fixation when they browse app interfaces with abundant information. For the case of browsing concise app interfaces, the operation level is not significantly relevant to fixation duration.

The findings in this study could provide useful information which can be taken into consideration during the design of apps and advertisements. Sample size is critical for the confidence of the findings in a study. Eighteen participants recruited in our study are not a large cohort. More participants are preferred to replicate the findings of the study.

## Data Availability

The dataset used in this study are available on request to the corresponding author.

## Footnotes

1 https://www.statista.com/statistics/276623/number-of-apps-available-in-leading-app-stores/.

2 https://www.statista.com/statistics/270291/popular-categories-in-the-app-store/.
